# INTRODUCING CENTENARIAN STUDIES IN AN AGING WORLD

**DOI:** 10.1093/geroni/igad104.1337

**Published:** 2023-12-21

**Authors:** Peter Martin

**Affiliations:** Iowa State University, Ames, Iowa, United States

## Abstract

Centenarian studies have come of age. With the first comprehensive centenarian studies conducted more than 35 years ago, there are now centenarian databases in the United States, in Europe, and in Asian countries. The major focus of these studies has been on genetic and family longevity factors, on support systems, personality, and on health behaviors. Another emphasis has been on various health components, such as physical health, mental health, frailty, and psychological well-being. In recent years, a second generation of centenarian studies has been initiated, comparing earlier born cohorts with later born cohorts. Among the first studies conducting a cohort comparison, the Danish centenarian studies, the Georgia centenarian studies, and the Tokyo centenarian studies showed inconsistent results. Whereas some studies indicated that later born cohorts enjoyed higher levels of functioning (e.g., Denmark, U.S. Georgia), other studies showed poorer performance (e.g., Japan). The current symposium sheds additional light on cohort comparisons with recently collected data from the second Hong Kong Centenarian Study, the Kyotango centenarian cohort, and the Health and Retirement Study. This presentation summarizes major findings from centenarian studies conducted around the world and highlights activities of the International Consortium of Centenarian Studies (ICC).

